# Mature Teratoma of the Cheek

**Published:** 2012-09-01

**Authors:** Mahavir Singh, KN Rattan, Babita Rani, Yogender Kadian, Sonia Hasija

**Affiliations:** Department of Pediatric Surgery, Pt.BD Sharma, Postgraduate Institute of Medical Sciences, Rohtak; Department of Pediatric Surgery, Pt.BD Sharma, Postgraduate Institute of Medical Sciences, Rohtak; Department of Community Medicine, Pt.BD Sharma, Postgraduate Institute of Medical Sciences, Rohtak; Department of Pediatric Surgery, Pt.BD Sharma, Postgraduate Institute of Medical Sciences, Rohtak; Department of Pathology, Pt.BD Sharma, Postgraduate Institute of Medical Sciences, Rohtak

A 2.5-kg girl was born with a growth inside the mouth associated with feeding difficulty. There was no respiratory difficulty. Antenatal ultrasound did not detect any abnormality. On examination a round, 3x2 cm wide base, uneven, lobulated, pink-colored soft tissue mass was protruding into the mouth. Precise tumor attachment could not be appreciated during the initial examination (Fig.1). 

**Figure:**
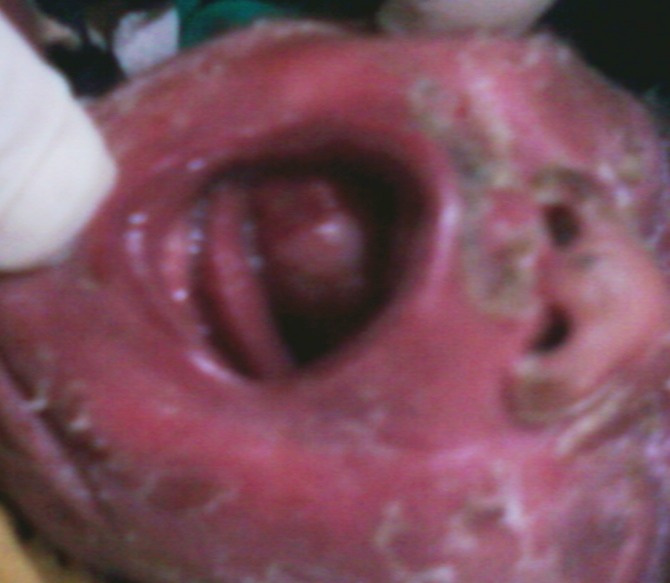
Figure 1: A round, 3x2 cm wide base, pink-colored soft tissue mass protruding into the mouth.

Mass was mixed in consistency with firm and cystic areas on palpation. Adjacent tissues appeared normal on examination. She was booked for excision which was done with an elliptical incision at the base of the lesion. Mucosal defect was left open. There was minimal blood loss. Postoperative recovery was uneventful. The neonate was discharged home on the following day. Excised tissue was sent for histopathological examination. The microscopic examination revealed mature teratoma with mature stratified squamous epithelium, mucinous gland and mesenchymal tissue scattered throughout (Fig. 2,3). A baseline alpha fetoprotein level was normal. Patient was advised for regular follow-up to detect any recurrence.

**Figure:**
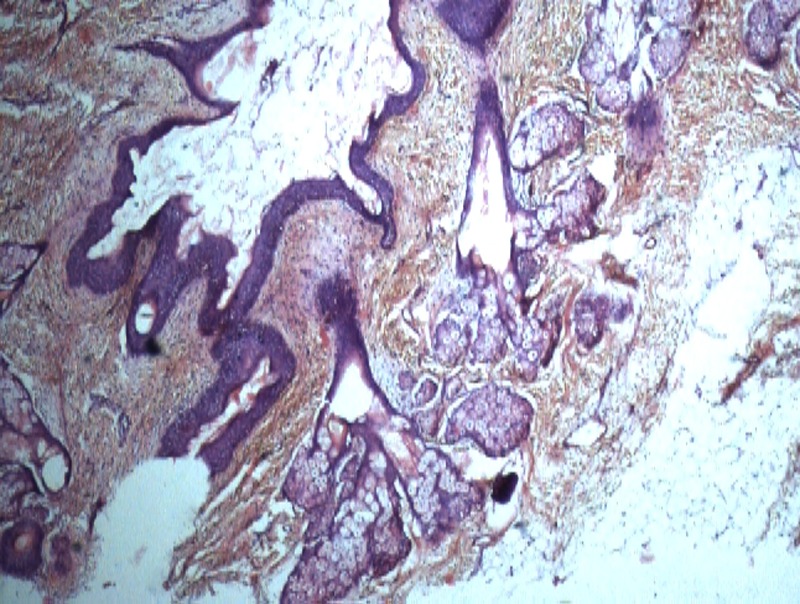
Figure 2: Photomicrograph showing stratified squamous epithelium along with cartilaginous tissue and mucosal gland. ( 200x HE).

**Figure:**
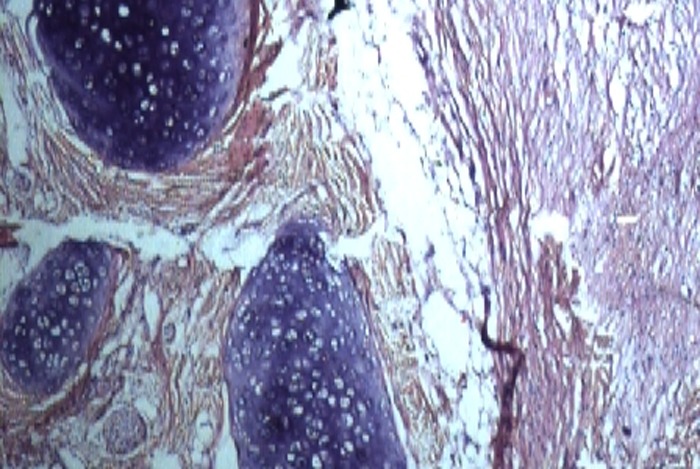
Figure 3: Micrograph showing mature teratoma having cartilaginous tissue along with mucinous glands.

## DISCUSSION

Oropharyngeal teratomas represent 2% of all teratomas. Epignathus is a term used to describe teratomas protruding from the mouth. These tumors arise from the soft or hard palates in the region of Rathke’s pouch. They generally fill the oral cavity and protrude out through the mouth. Pharyngeal teratomas arise from the posterior aspect of the nasopharynx. Cheek is the least mentioned site of teratoma in literature. Even after extensive search we could retrieve only three cases of teratoma arising from cheek or buccal mucosa [1-3]. 

Although most of these tumors are benign, they frequently cause significant airway and esophageal obstruction in the perinatal period and thus potentially fatal. Large tumors can interfere with fetal swallowing and produce polyhydramnios, cause severe respiratory distress at birth, and may lead to stillbirth [4]. 

Prenatal ultrasound is a reliable diagnostic tool for detecting these lesions as early as 21 week gestation, thereby allowing for careful arrangement of the time, mode, and place of delivery. Approximately one third of prenatally diagnosed cases are complicated by maternal polyhydramnios resulting in high incidence of preterm labor and delivery. Serial amnio-reduction may be required to prevent this complication [5,6]. 

At birth, death may result from tracheal compression or deviation and inability to intubate. Prenatal diagnosis permits cesarean section and establishment of an airway before the cord is clamped. This is done by employing an EXIT (ex utero intrapartum treatment) procedure. Because precipitous airway obstruction may occur due to hemorrhage inside the tumor, tracheal intubation is indicated in all patients, regardless of the presence or absence of symptoms [5-7].

## Footnotes

**Source of Support:** Nil

**Conflict of Interest:** None declared
